# Glucose-6-Phosphate Dehydrogenase Deficiency and Physical and Mental Health until Adolescence

**DOI:** 10.1371/journal.pone.0166192

**Published:** 2016-11-08

**Authors:** Man Ki Kwok, Gabriel M. Leung, C. Mary Schooling

**Affiliations:** 1 School of Public Health, Li Ka Shing Faculty of Medicine, The University of Hong Kong, Hong Kong Special Administrative Region, China; 2 City University of New York Graduate School of Public Health and Health Policy, New York, New York, United States of America; Mayo Clinic Arizona, UNITED STATES

## Abstract

**Background:**

To examine the association of glucose-6-phosphate dehydrogenase (G6PD) deficiency with adolescent physical and mental health, as effects of G6PD deficiency on health are rarely reported.

**Methods:**

In a population-representative Chinese birth cohort: “Children of 1997” (n = 8,327), we estimated the adjusted associations of G6PD deficiency with growth using generalized estimating equations, with pubertal onset using interval censored regression, with hospitalization using Cox proportional hazards regression and with size, blood pressure, pubertal maturation and mental health using linear regression with multiple imputation and inverse probability weighting.

**Results:**

Among 5,520 screened adolescents (66% follow-up), 4.8% boys and 0.5% girls had G6PD deficiency. G6PD-deficiency was not associated with birth weight-for-gestational age or length/height gain into adolescence, but was associated with lower childhood body mass index (BMI) gain (-0.38 z-score, 95% confidence interval (CI) -0.57, -0.20), adjusted for sex and parental education, and later onset of pubic hair development (time ratio = 1.029, 95% CI 1.007, 1.050). G6PD deficiency was not associated with blood pressure, height, BMI or mental health in adolescence, nor with serious infectious morbidity until adolescence.

**Conclusions:**

G6PD deficient adolescents had broadly similar physical and mental health indicators, but transiently lower BMI gain and later pubic hair development, whose long-term implications warrant investigation.

## Introduction

Glucose-6-phosphate dehydrogenase (G6PD) deficiency is the most common enzyme deficiency worldwide affecting 400 million people mainly from Sub-Saharan Africa, the Mediterranean and Southeast Asia with prevalence ranging from 3% to 26%.[1] G6PD is X-linked and recessive so boys are more susceptible than girls; nonetheless, some girls with heterozygous genes may also be affected due to randomly occurring unequal inactivation of the X-chromosome.[2] Low G6PD affects red blood cells because their defense against oxidative damage relies heavily on G6PD.[2] G6PD deficiency causes neonatal jaundice and acute hemolytic anemia on exposure to fava beans, infections or certain medications.[2] G6PD deficiency is thought to confer protection against malaria,[1] whether such survival trades-off against any other aspects of fitness[3] is unknown. G6PD catalyses the initiation of the pentose phosphate pathway generating nicotinamide adenine dinucleotide phosphate (NADPH), so G6PD deficiency limits NADPH production and may inhibit NADPH-dependent pathways. Specially, G6PD deficiency could inhibit the 3-hydroxy-3-methylglutaryl-coenzyme A (HMG-CoA) reductase used for cholesterol synthesis,[4, 5] or cytochrome P450 enzymes used for steroid metabolism,[6] when the HMG-CoA reductase inhibitor, statins, and HMG-CoA reductase genetic variants, protect against cardiovascular disease[7] and impair glucose metabolism.[8] G6PD deficiency can also result in hyperbilirubin which could cause brain damage and dysfunction.[9] G6PD deficiency has been reported as associated with a higher risk of diabetes in a large cross-sectional study from Israel using medical records[10] and several small hospital-based case-control studies,[10–14] although not in a small study from Italy.[15] G6PD deficiency has also been associated with lower risk of cardiovascular disease mortality in two cross-sectional studies from Sardinia (Italy),[16, 17] and with lower risk of myocardial infarction in a case-control study,[4] although associations of G6PD deficiency with cardiovascular disease risk factors[18, 19] and all-cause mortality[17, 20] are inconsistent. G6PD deficiency has also been linked with mental illnesses in adults in some case reports/series,[21, 22] and genetic linkage studies[23–25] but not all.[26–30] Genetic studies are less open to confounding than most observational studies, nevertheless the evidence concerning effects of G6PD is limited and potentially biased by design flaws, such as inappropriate selection of controls in case-control studies. No studies have systematically assessed how G6PD deficiency affects health across the life-course, especially from an evolutionary biology perspective that early life survival (from malaria infections)[1] might trade-off against reproduction and/or growth and development.[3] Here, we examined the associations of G6PD deficiency with key aspects of physical and mental health from birth to adolescence including size and growth, blood pressure, and mental health (as proxies of growth and development), puberty (as a proxy of reproductive success) and serious infections (as a proxy of immune system maintenance) using a large, population-representative Chinese birth cohort: “Children of 1997” in Hong Kong this ethnically homogeneous (>95%) Chinese population with a universal (99% coverage) neonatal G6PD screening programme.[31]

## Materials and Methods

### Data Source

Hong Kong’s “Children of 1997” birth cohort is a population representative Chinese birth cohort (*n* = 8,327) that covered 88.0% of all births in Hong Kong from April 1, 1997 to May 31, 1997, described in detail elsewhere.[32] The study was initially established to investigate the effect of secondhand smoke exposure on infant health. Families were recruited at the first postnatal visit to any of the 49 Maternal and Child Health Centers (MCHCs) in Hong Kong, which parents of all newborns are strongly encouraged to attend for free vaccinations and well-baby checks. Characteristics obtained using a self-administered questionnaire in Chinese at recruitment and subsequent routine visits include maternal and birth characteristics and socioeconomic position (SEP). Passive follow-up via record linkage was instituted in 2005 to obtain weight and height from birth to 5 years and birth characteristics, including G6PD status, from the MCHCs (*n* = 7,999, 96% successful matching); annual weight and height and bi-annual pubertal status (grade 1 (age 6–7 years) onwards), blood pressure (grade 5 (age 10–11 years) onwards), emotional and behavioral problems (grade 2, 4 and 6 (i.e., ages 7–8, 9–10 and 11–12 years) and self-esteem (grade 4 (age 9–10 years) onwards) from the Student Health Service, Department of Health, which provides free annual check-ups for all school students (*n* = 7,809, 94% successful matching); and hospital admission records with details on principal diagnosis code, date of admission and discharge from the Hospital Authority, which provides in-patient services at minimal cost accounting for 90% of total bed days (*n* = 7,352, 88% successful matching).[33] Admissions are coded at discharge according to the *International Classification of Diseases*, *Ninth Version Clinical Modification* (ICD-9CM). At the Student Health Service, height was measured by stadiometer and weight by digital scale. Blood pressure was measured using an automated oscillometric device, with initial values over the 90^th^ percentile reference re-checked with a sphygmomanometer and recorded. Pubertal status for breast or genitalia and pubic hair development was clinically assessed according to the criteria of Marshall and Tanner and testicular volume assessed by orchidometer. Emotional and behavioral problems were assessed from the Revised Parent’s Rutter Scales,[34] self-esteem from the Form A of the Culture-Free Self Esteem Inventories,[35] and depressive symptoms from the Patient Health Questionnaire-9 (PHQ-9), validated in Chinese adolescents,[36] in Survey II (2010–2012) and pilots for in-person follow-up.

### G6PD status

G6PD status was categorized as “G6PD-deficient” or “non-G6PD-deficient” based on the MCHC record. In Hong Kong, a free-of-charge universal neonatal screening program assesses erythrocyte G6PD activity from umbilical cord blood at birth.[31] Parents of newborns with G6PD activity below 25% of the mean are informed and followed-up by the Clinical Genetic Service of the Department of Health,[31] who confirm G6PD status based on whole blood specific enzyme activity testing.

### Outcomes

#### Growth and size

Growth outcomes considered included birth weight, growth rate in infancy, childhood and at puberty as well as height and BMI at ~15 years. We used birth weight-for-gestational age z-score relative to sex- and gestational age-specific contemporary Hong Kong Chinese infants to proxy fetal growth.[37] We used length/height or BMI gain z-scores during 3 to 9 months for infancy (birth-<2 years) relative to the 2005 WHO growth standards for 0–5 years[38] and 3 to 7 years for childhood (2-<8 years) and 8 to 15 years for puberty (8-<16 years) relative to the 2007 WHO growth references for 5–19 years.[39] Each cohort member had up to 13 length/height or BMI measurements taken at about 3 and 9 months for infancy, at 3, 6 and 7 years for childhood, and at 8, 9, 10, 11, 12, 13, 14 and 15 years for puberty. Since length/height or BMI reflects an accumulation of prior exposures, we included initial size at each growth phase (birth weight z-score for infancy, height or BMI z-score at 9 months for childhood and height or BMI z-score at 7 years for puberty) to identify gain at each growth phase. Finally, we considered height and BMI z-scores at ~15 years (14-<16 years).

#### Puberty

Pubertal outcomes considered were age at onset of puberty, age of menarche and testicular volume. Age at pubertal onset was defined, as the earliest age when Tanner stage II for breast or genitalia and pubic hair was recorded. Children with infeasible sequences of pubertal stages, such as pubertal stage II before pubertal stage I were excluded.

#### Blood pressure

Systolic and diastolic blood pressure at ~11 (based on the closest measurement available from 9-<12 years) and 13 years (12-<15 years) z-scores relative to age-, sex- and height-standardized blood pressure standards from the United States National High Blood Pressure Education Group in 2004[40] were used.

#### Serious infections

Hospitalizations (including same-day discharge and in-patient admission for at least 24 hours) for respiratory (and related) infections (principal diagnosis of ICD-9CM 33, 34.0, 381–2, 460–6, 477, 480–7, 477 or 493), gastrointestinal infections (ICD-9CM 001–009, 535.00, 535.50, 558.9, 538, 535.40 or 787.91) and other infections (ICD-9CM 10–32, 34.1–139, 320–1, 370, 372.0–372.3, 390–2, 540–2, 590, 595, 599.0, 680–6, 771, 780.3, or 780.6) were considered. Since inclusion of hospitalizations in the immediate neonatal period could have introduced bias given the average hospital stay for infants delivered by caesarean section (7.9 days) differed from natural birth (3.0 days), time to first admission was considered from the first 9 days of life until 12.0 years of age. We also considered infections by age group (i.e., 9 days-6 years and >6–12 years) to reflect diet transitions, motor development and increasing social contact.

#### Mental health

Mental health was assessed from Rutter score, self-esteem and PHQ9. Parent-reported Rutter scores at ~11 years (based on the closest measurement available from 9-<12 years) consist of 31 items describing emotional and behavioral difficulties, with each item scored 0 for does not apply, 1 for applies somewhat or 2 for certainly applies. A total score and subscores for conduct problems (5 items), emotional problems (5 items) and inattention/hyperactivity (3 items) were calculated, where a higher score indicated more emotional and behavioral problems. Self-reported self-esteem scores at ~11 years (9-<12 years) consist of a total score and subscores for general (perception of self-worth in general) (20 items), social (perception of quality of relationships with peer) (10 items), academic (perception of ability to academic success) (10 items), and parent-related (perception of status at home) (10 items) self-esteem were calculated, where a lower score indicated lower self-esteem. Self-reported PHQ-9 scores at ~13 years (12-<15 years) consist of 9 items describing symptoms and functional impairment, with each item scored 0 for not at all, 1 for several days, 2 for more than half the days or 3 for nearly every day. A total PHQ-9 score was calculated, where a higher score indicated more depressive symptoms.

### Statistical analysis

We assessed the adjusted associations of G6PD deficiency with birth weight-for-gestational age z-score, length/height and BMI z-scores gain during infancy, childhood, and puberty using generalized estimating equations with an exchange working correlation structure adjusted for initial size. We assessed the adjusted associations of G6PD deficiency with age at pubertal onset using interval-censored regression,[41] using a log-normal distribution, from which a time ratio greater than 1 indicates older age at pubertal onset, while a time ratio less than 1 indicates younger age at pubertal onset. We assessed the adjusted associations of G6PD deficiency with age at menarche, testicular volume, blood pressure at ~11 or ~13 years, height and BMI z-scores at ~15 years using linear regression. We assessed the adjusted associations of G6PD deficiency with Rutter score, self-esteem score and depressive symptom score using negative binomial regression. We assessed the adjusted associations of G6PD deficiency with time to first hospitalization for respiratory infections, gastro-intestinal infections, other infections and all infections up until 12 years using Cox proportional hazard regression.

We compared baseline characteristics between those with and without G6PD status using Cohen effect sizes. Confounders included were sex and highest parental education; race/ethnicity was not considered because all cohort participants are Chinese. We used inverse probability weighting with multiple imputation (IPW/MI) to recover the representativeness of entire sample based on the complete cases[42] thereby accounting for any selection bias due to missing G6PD status. We used multiple imputation for confounders, although we had <10% missing for each confounder, based on a flexible additive regression model with predictive mean matching[43] incorporating data on the exposure, outcomes, confounders, and other covariates (sex, mode of delivery, birth order, secondhand smoke exposure, type of hospital at birth, mother’s and father’s age, mother’s and father’s birthplace, parental education, household income, housing).[44] We created 20 imputed datasets and combined them taking into account variability between and within imputations based on Rubin’s formula.[43] We predicted the probability of missing G6PD status using logistic regression based on these confounders and covariates (sex, mode of delivery, secondhand smoke exposure, type of hospital at birth, mother’s birthplace, parental education, household income, housing) to generate the inverse probability weights. We used weighted regression models, from which sandwich variance estimators accounting for the weights were presented. A sample R code for implementing the IPW/MI is available in the S1 Appendix. Among 5,520 adolescents with G6PD status, we performed an available case analysis, i.e., deleting cases with missing data on variables on an analysis-by-analysis basis, for each health outcomes. Since G6PD deficiency is more common in boys, as a sensitivity analysis, we also re-examined the associations among 2,949 boys.

#### Power analysis

Given 153 children (91.5% boys) were G6PD-deficient in this birth cohort, we estimated the minimum effect size that can be detected with 80% power and level of significance at 0.05. Our study would allow detection of a mean difference of 0.32 in BMI z-scores, or 0.61 kg/m^2^ in BMI during childhood.

Statistical analyses were performed using Stata version 10 (Stata Corp, College station, Texas, USA) and R version 3.0.1 (R Development Core Team, Vienna, Austria).

### Ethics approval

Since our participants are children, informed (non-written) consent for the original survey and subsequent record linkage was obtained from the parents, next of kin, caretakers or guardians (informants) on behalf of the participants by the informant agreeing and subsequently completing the questionnaire at enrollment, this manner of obtaining consent was approved by The University of Hong Kong Medical Faculty Ethics Committee over 20 years ago. Informed written consent for subsequent Surveys and in-person follow up was obtained from a parent or guardian, or at ages 18+ years from the participant. Ethical approval for all studies, including passive follow-up via record linkage, was obtained from the University of Hong Kong-Hospital Authority Hong Kong West Cluster Joint Institutional Review Board and also, where appropriate, the Ethics Committee of the Department of Health, Government of the Hong Kong Special Administrative Region.

## Results

Of the original 8,327 cohort members, as of January 2014, 27 had permanently withdrawn. The 2,780 adolescents missing G6PD status did not differ from the other 5,520 in terms of sex, mode of delivery, secondhand smoke exposure, mother’s birthplace, parental education, household income and housing, but were more likely to be born in public hospitals (S1 Table). Among the 5,520 adolescents with G6PD status (66% follow-up), 140 (4.8%) boys and 13 (0.5%) girls were G6PD-deficient with a total 72,889 person-years of follow-up (mean 13.2 years, maximum 15.9 years). Table 1 shows that G6PD deficiency was associated with lower parental education but not with mode of delivery, secondhand smoke exposure, type of hospital at birth, mother’s birthplace, household income or type of housing. G6PD deficiency was also associated with higher risk of hospital admission for neonatal jaundice (hazard ratios 2.49, 95% confidence interval (CI) 1.19, 5.18) as would be expected.

**Table 1:** Baseline characteristics by G6PD status for adolescents from Hong Kong’s “Children of 1997” birth cohort, Hong Kong, China, 1997–2010.

	G6PD status				
	Deficient		Non-deficient		
	(*n* = 153)		(*n* = 5,367)		
Characteristics	No.	%	No.	%	P-value
Child’s sex					<0.001
Female	13	8.5	2,557	47.7	
Male	140	91.5	2,809	52.4	
Mode of delivery					0.38
Natural labour	70	48.3	2,774	53.8	
Assisted natural labour	30	20.7	899	17.5	
Caesarean birth	45	31.0	1,479	28.7	
Secondhand smoke exposure					0.43
None	40	28.0	1,443	28.4	
Non-parental household smoking	52	36.4	1,964	38.7	
Paternal smoking	40	28.0	1,428	28.1	
Maternal smoking	11	7.7	239	4.7	
Type of hospital at birth					0.75
Public	103	67.8	3,546	66.5	
Private or overseas	49	32.2	1,783	33.5	
Mother’s birthplace					0.16
Mainland China or elsewhere	61	42.1	1,865	36.4	
Hong Kong	84	57.9	3,257	63.6	
Highest parental education at recruitment					0.01
Grade 9 or below	55	37.2	1,478	28.3	
Grade 10–11	64	43.2	2,229	42.6	
Grade 12 or above	29	19.6	1,521	29.1	
Household income per head at recruitment ^a^					0.12
1^st^ quintile	38	26.8	889	18.3	
2^nd^ quintile	25	17.6	930	19.2	
3^rd^ quintile	28	19.7	962	19.8	
4^th^ quintile	23	16.2	1,023	21.1	
5^th^ quintile	28	19.7	1,053	21.7	
Type of housing at recruitment					0.35
Public estate	68	46.9	2,244	43.7	
Subsidized home ownership flat	27	18.6	824	16.0	
Private flat	50	34.5	2,072	40.3	

^a^ Mean (standard deviation) for household income per head at recruitment in quintiles (in Hong Kong dollar; pegged at a rate of 7.8 dollar = 1 U.S. dollar) were 1^st^ quintile: $1,751 (414), 2^nd^ quintile: $2,849 (319), 3^rd^ quintile: $4,359 (557), 4^th^ quintile: $6,827 (890) and 5^th^ quintile: $15,287 (17,613).

Table 2 shows that G6PD deficiency was not associated with birth weight-for-gestational age z-score or length/height z-score gain from infancy to adolescence, but was associated with lower childhood BMI z-score gain, adjusted for sex and parental education. G6PD deficiency was associated with later onset of pubic hair development, but not with breast or genitalia development, age at menarche or testicular volume. G6PD was not associated with systolic or diastolic blood pressure z-scores at ~11 or ~13 years or with height or BMI z-scores at ~15 years. Table 3 shows that G6PD deficiency was not associated with hospital admission for respiratory infections, gastro-intestinal infections, or other infections up until 12 years. Table 4 shows that G6PD deficiency was not associated with behavioral or emotional problems at ~11 years, self-esteem at ~11 years or depressive symptoms at ~13 years. The sensitivity analysis produced similar patterns of associations among boys, and also indicated lower pubertal height z-score gain (S2–S4 Tables).

**Table 2:** Adjusted^a^ associations of G6PD status with birth weight-for-gestational age z-score for growth during fetal phase, height and body mass index (BMI) gain z-scores during infancy, childhood and pubertal phases, age at onset of breast or genitalia or pubic hair development (Tanner stage II), testicular volume, age at menarche and blood pressure at ~11 and ~13 years, height and BMI z-scores at ~15 years in the Hong Kong’s “Children of 1997” birth cohort, Hong Kong, China, 1997–2010.

Age	Outcomes	G6PD status	n	Mean difference^c^	95% CI	Follow-up n (%)
Fetal	Birth weight-for-	Deficient	153	-0.05	-0.21, 0.11	5,513
	gestational age z-score	Non-deficient	5,360	Reference		(99.9)
Infancy	Length gain z-score^b^	Deficient	126	-0.01	-0.15, 0.13	4,562
		Non-deficient	4,436	Reference		(82.6)
	BMI gain z-score^b^	Deficient	126	0.01	-0.18, 0.19	4,562
		Non-deficient	4,436	Reference		(82.6)
Childhood	Height gain z-score^b^	Deficient	139	-0.03	-0.15, 0.09	5,041
		Non-deficient	4,902	Reference		(91.3)
	BMI gain z-score^b^	Deficient	139	-0.38	-0.57, -0.20	5,041
		Non-deficient	4,902	Reference		(91.3)
Puberty	Height gain z-score^b^	Deficient	143	-0.04	-0.10, 0.01	5,158
		Non-deficient	5,015	Reference		(93.4)
	BMI gain z-score^b^	Deficient	143	-0.05	-0.16, 0.07	5,158
		Non-deficient	5,015	Reference		(93.4)
			n	Time ratio	95% CI	
	Age at onset of breast or	Deficient	136	1.010	0.987, 1.030	5,105
	genitalia development	Non-deficient	4,969	1.000		(92.5)
	Age at onset of pubic	Deficient	138	1.029	1.007, 1.050	5,129
	hair development	Non-deficient	4,991	1.000		(92.9)
	Age at onset of testes	Deficient	124	1.008	0.988, 1.030	2,743
	development	Non-deficient	2,619	1.000		(93.0)
			n	Mean difference^c^	95% CI	
	Testicular size	Deficient	125	-0.22	-0.74, 0.30	2,763
		Non-deficient	2,638	Reference		(93.7)
	Age at menarche	Deficient	12	-0.36	-0.94, 0.22	1,951
		Non-deficient	1,939	Reference		(75.9)
11 years	Systolic blood pressure	Deficient	124	-0.001	-0.17, 0.17	4,771
	z-score	Non-deficient	4,347	Reference		(81.0)
	Diastolic blood pressure	Deficient	124	0.05	-0.05, 0.14	4,771
	z-score	Non-deficient	4,347	Reference		(81.0)
13 years	Systolic blood pressure	Deficient	99	0.01	-0.20, 0.21	3,871
	z-score	Non-deficient	3,772	Reference		(70.1)
	Diastolic blood pressure	Deficient	99	-0.03	-0.14, 0.08	3,871
	z-score	Non-deficient	3,772	Reference		(70.1)
15 years	Height z-score	Deficient	78	-0.09	-0.27, 0.10	3,123
		Non-deficient	3,045	Reference		(56.6)
	BMI z-score	Deficient	78	-0.09	-0.36, 0.17	3,123
		Non-deficient	3,045	Reference		(56.6)

^a^ Adjusted for sex and highest parental education

^b^ Additionally adjusted for initial size (birth weight z-score for infancy, height or BMI z-score at 9 months for childhood phase, height or BMI z-score at 7 years for pubertal phase)

^c^ Mean difference in z-score: 1 unit change in birth weight-for-gestational age z-score is approximated to 370 grams; 1 unit change in height z-score is approximated to 2.3 cm at 9 months, 5.6 cm at 7 years and 7.4 cm at 13 years; 1 unit change in body mass index z-score is approximated to 1.5 kg/m^2^ at 9 months, 1.9 kg/m^2^ at 7 years and 2.7 kg/m^2^ at 13 years; 1 unit change in systolic blood pressure z-score is approximated to 10.6 mmHg and 1 unit change in diastolic blood pressure z-score is approximated to 11.3 mmHg.

**Table 3:** Adjusted^a^ associations of G6PD status with time to first hospitalization for respiratory infections, gastrointestinal infections, other (non-respiratory or non-gastrointestinal) infections and all infections up until 12 years by age group in the Hong Kong’s “Children of 1997” birth cohort, Hong Kong, China, 1997–2010.

Age	Hospitalization	G6PD status	Case no.	Hazard ratios	95% CI
			n (%)		
9 days to 12 years					
	Respiratory infections	Deficient	37 (24.2%)	0.84	0.63, 1.11
		Non-deficient	1,368 (25.5%)	1.00	
	Gastrointestinal infections	Deficient	17 (11.1%)	0.86	0.57, 1.30
		Non-deficient	613 (11.4%)	1.00	
	Other infections	Deficient	17 (11.1%)	0.85	0.57, 1.29
		Non-deficient	600 (11.2%)	1.00	
	All infections	Deficient	56 (36.6%)	0.88	0.70, 1.11
		Non-deficient	1,992 (37.1%)	1.00	
9 days to 6 years					
	Respiratory infections	Deficient	32 (20.9%)	0.78	0.58, 1.06
		Non-deficient	1,262 (23.5%)	1.00	
	Gastrointestinal infections	Deficient	16 (10.5%)	0.86	0.56, 1.32
		Non-deficient	574 (10.7%)	1.00	
	Other infections	Deficient	16 (10.5%)	0.88	0.57, 1.34
		Non-deficient	542 (10.1%)	1.00	
	All infections	Deficient	52 (34.0%)	0.88	0.69, 1.11
		Non-deficient	1,855 (34.6%)	1.00	
6 to <12 years					
	Respiratory infections	Deficient	8 (5.2%)	1.30	0.69, 2.46
		Non-deficient	201 (3.8%)	1.00	
	Gastrointestinal infections	Deficient	3 (2.0%)	1.00	0.30, 3.30
		Non-deficient	52 (1.0%)	1.00	
	Other infections	Deficient	2 (1.3%)	1.26	0.35, 4.52
		Non-deficient	69 (1.3%)	1.00	
	All infections	Deficient	13 (8.5%)	1.18	0.72, 1.93
		Non-deficient	303 (5.7%)	1.00	

^a^ Adjusted for sex, highest parental education and proxies of preferred service sector (type of hospital at birth and household income per head).

**Table 4:** Adjusted^a^ association of G6PD status with mental health indicated by mean difference in Rutter score at ~11 years, self-esteem score at ~11 years and depressive symptoms score at ~13 years in the Hong Kong’s “Children of 1997” birth cohort, Hong Kong, China, 1997–2010.

Age	Outcomes	G6PD status	n	Mean difference	95% CI	Follow-up n (%)
11 years	**Rutter score**					3,809
	Total	Deficient	107	-0.05	-0.17, 0.08	(69.0)
		Non-deficient	3,702	Reference		
	Conduct	Deficient	107	0.02	-0.13, 0.17	
		Non-deficient	3,702	Reference		
	Emotional	Deficient	107	-0.03	-0.20, 0.13	
		Non-deficient	3,702	Reference		
	Hyperactivity	Deficient	107	-0.17	-0.35, 0.02	
		Non-deficient	3,702	Reference		
11 years	**Self-esteem score**					4,700
	Total	Deficient	128	0.01	-0.03, 0.04	(85.1)
		Non-deficient	4,572	Reference		
	General	Deficient	128	0.003	-0.04, 0.04	
		Non-deficient	4,572	Reference		
	Social	Deficient	128	-0.01	-0.07, 0.05	
		Non-deficient	4,572	Reference		
	Academic	Deficient	128	0.02	-0.04, 0.08	
		Non-deficient	4,572	Reference		
	Parent-related	Deficient	128	0.01	-0.04, 0.06	
		Non-deficient	4,572	Reference		
13 years	**Depressive symptoms**					3,932
	PHQ-9 score	Deficient	111	0.04	-0.15, 0.22	(71.2)
		Non-deficient	3,821	Reference		

^a^ Adjusted for sex, highest parental education, age at measurement and survey mode (for PHQ-9).

## Discussion

In this contemporary, population-representative Hong Kong Chinese birth cohort with extensive longitudinal measurements of physical and mental health across key life stages for growth and development, G6PD deficiency was associated with transiently lower childhood BMI gain and later onset of pubic hair development. Otherwise, G6PD-deficient adolescents had similar current size, blood pressure, risk of serious infectious morbidity requiring hospitalization, emotional or behavioral problems, self-esteem or depressive symptoms as others. Results among boys only were similar. Whether the difference in childhood size gain and pubertal development by G6PD status is transient or an indicator of longer term health has yet to be elucidated. Our findings highlight the need for better understanding of G6PD deficiency across the life course for the large numbers globally with G6PD.

Despite using prospectively collected height and weight, clinically assessed pubertal development, routinely measured blood pressure, physician-diagnosed hospital admission records and parent- and self-reported psychological outcomes, limitations exists. First, we do not have erythrocyte G6PD activity, thus cannot assess any graded association with health outcomes. The prevalence of G6PD deficiency in our study is the level expected in our population,[31] so we cannot detect very small differences that might be important at a population level even if not clinically meaningful. Second, population stratification could introduce bias when studying the associations of G6PD with health across subgroups with different genetic ancestry. Within an ethnically homogeneous Chinese birth cohort whose parents and grandparents largely originate from Hong Kong or the neighbouring province of Guangdong, confounding by population stratification should be minimal in our setting. Third, although missing G6PD status is unlikely to differ by child health outcomes, random misclassification would make our results conservative. Given missing G6PD status was more common among those born in public hospitals, a particular set of characteristics may be under-represented in the included participants. We used IPW/MI to account for the inclusion probability by assigning a larger weight to those similar to the excluded based on infant characteristics, family SEP, mother’s birthplace and a proxy of preferred service use so as to be recover the original sample. Fourth, private hospital admissions are lacking, perhaps mostly likely for families of higher SEP. However, the associations of G6PD status with hospitalization did not differ by indicators of preferred service sector. Our findings would be biased if the association of G6PD with hospitalization differed among private hospital users, which is unlikely. To account for possible confounding, we adjusted for these indicators, as adjustment means examining the association separately for public hospital users and private hospital users before taking the average. Fifth, follow-up of our cohort was not complete for health outcomes at different ages, although inclusion of adolescents with particular combinations of G6PD deficiency and growth, size, pubertal development, blood pressure, time to hospitalization and psychological outcomes is unlikely. Sixth, given five different traits considered here, as a sensitivity analysis, we re-checked the associations with adjustment for multiple comparisons, using a Bonferroni corrected significance level of 0.01 (0.05/5) to account for testing 5 traits. G6PD-deficiency remained associated with lower childhood BMI gain (-0.38 z-score, 99% CI -0.64, -0.15) and later onset of pubic hair development (time ratio = 1.029, 99% CI 1.000, 1.058), adjusted for sex and parental education. Finally, we are limited by the age of the cohort, so we could not consider cardiovascular disease and diabetes as outcomes. Whether the effect of G6PD deficiency on chronic diseases could become more evident in adulthood requires future follow-up. We also did not assess the known link of G6PD deficiency with acute hemolytic anemia because this birth cohort is population-representative and hence has insufficient cases of serious complications for a relatively rare condition such as G6PD.

This is the first study addressing G6PD deficiency and health beyond early infancy. The null association of G6PD deficiency with emotional and behavioral problems, self-esteem and depressive symptoms in late childhood and early adolescence, together with the conflicting findings from case reports/series of adult psychiatric patients or genetic linkage studies[21–30] suggests G6PD deficiency is unlikely to cause mental health problems. Further follow-up of adult mental health and replication in other settings would provide even more reassurance. G6PD deficiency was not associated with adolescent blood pressure or size, consistent with our previous finding that blood pressure is more associated with pubertal rather than earlier size[45] and with no association of G6PD status with adult blood pressure observed elsewhere.[19] Lower childhood BMI gain may simply delay pubertal onset. However, G6PD deficiency affects the NADPH pathway that is required for cytochrome P450 enzymes to metabolize steroids,[6] consistent with our findings of specifically later pubic hair development driven by androgens[46] in G6PD-deficient boys. Earlier adrenarche is associated with higher blood erythrocyte count and higher hemoglobin.[47] Whether reduced erythrocyte metabolism in G6PD deficiency[48] could be partly due to lower adrenal androgens has not been previously been considered. Nevertheless such a hypothesis would provide an underlying explanation for G6PD deficiency, or other exposures, such as statins, having different associations with cardiovascular disease[4, 16, 17] and diabetes,[10–14] because androgens have different effects on cardiovascular disease[49] and glucose metabolism,[50, 51] as well as potentially representing the evolutionary biology trade-off of survival (here from malaria) against reproductive success consistent with life-history theory.[3]

## Conclusions

In an economically developed setting, reassuringly G6PD deficiency had little impact on a range of physical and mental health indicators from birth to adolescence, although an association with lower childhood BMI and later pubic hair development in boys is possible. Whether such a difference is transient and whether G6PD deficiency affects health, particularly non-communicable diseases, in adulthood requires large-scaled prospective cohort studies with reliable G6PD status. Our findings would be useful to formulate evidenced-based advices for re-assuring parents that G6PD deficiency has minimal effects on many health outcomes in childhood. Equally important, this study helps generate discussion among clinical and public health practitioners concerning the lifelong health impact of G6PD deficiency.

## Supporting Information

S1 Appendix R sample code for inverse probability weighting with multiple imputation (IPW/MI). (DOCX)

S1 Table Baseline characteristics of 5,520 adolescents who were included in the analyses and 2,780 who were excluded from the analyses because of missing G6PD status in Hong Kong’s “Children of 1997” birth cohort, Hong Kong, China, 1997–2010. (DOCX)

S2 Table Adjusted association of G6PD status with birth weight-for-gestational age z-score for growth during fetal phase, height and body mass index (BMI) gain z-scores during infancy, childhood and pubertal phases, age at onset of breast or genitalia or pubic hair development (Tanner stage II), testicular volume, age at menarche and blood pressure at ~11 and ~13 years, height and BMI z-scores at ~15 years among boys in the Hong Kong’s “Children of 1997” birth cohort, Hong Kong, China, 1997–2010. (DOCX)

S3 Table Adjusted association of G6PD status with time to first hospitalization for respiratory infections, gastrointestinal infections, other (non-respiratory or non-gastrointestinal) infections and all infections up until 12 years by age groups among boys in the Hong Kong’s “Children of 1997” birth cohort, Hong Kong, China, 1997–2010. (DOCX)

S4 Table Adjusted association of G6PD status with mental health indicated by mean difference in Rutter score at ~11 years, self-esteem score at ~11 years and depressive symptoms score at ~13 years among boys in the Hong Kong’s “Children of 1997” birth cohort, Hong Kong, China, 1997–2010. (DOCX)

## Abbreviations

BMI: body mass index
CI: confidence interval
HMG-CoA: 3-hydroxy-3-methylglutaryl-coenzyme A
G6PD: glucose-6-phosphate dehydrogenase
MCHC: Maternal and Child Health Center
NADPH: nicotinamide adenine dinucleotide phosphate
SEP: socioeconomic position
WHO: World Health Organization
z-score: standard deviation score

## References

[1] Cappellini MD, Fiorelli G. Glucose-6-phosphate dehydrogenase deficiency. Lancet. 2008;371(9606):64–74. 10.1016/S0140-6736(08)60073-2 . 18177777

[2] WHO Working Group. Glucose-6-phosphate dehydrogenase deficiency. Bulletin of the World Health Organization. 1989;67(6):601–11. 2633878 PMC2491315

[3] Worthman CM, Kuzara J. Life history and the early origins of health differentials. Am J Hum Biol. 2005;17(1):95–112. 10.1002/ajhb.20096 . 15611966

[4] Meloni L, Manca MR, Loddo I, Cioglia G, Cocco P, Schwartz A, et al Glucose-6-phosphate dehydrogenase deficiency protects against coronary heart disease. Journal of inherited metabolic disease. 2008;31(3):412–7. 10.1007/s10545-008-0704-5 . 18392752

[5] Muntoni S, Muntoni S. Gene-nutrient interactions in G6PD-deficient subjects—implications for cardiovascular disease susceptibility. Journal of nutrigenetics and nutrigenomics. 2008;1(1–2):49–54. 10.1159/000109874 . 19918114

[6] Stanton RC. Glucose-6-phosphate dehydrogenase, NADPH, and cell survival. IUBMB life. 2012;64(5):362–9. 10.1002/iub.1017 22431005 PMC3325335

[7] Reriani MK, Dunlay SM, Gupta B, West CP, Rihal CS, Lerman LO, et al Effects of statins on coronary and peripheral endothelial function in humans: a systematic review and meta-analysis of randomized controlled trials. Eur J Cardiovasc Prev Rehabil. 2011;18(5):704–16. 10.1177/1741826711398430 . 21450596

[8] Swerdlow DI, Preiss D, Kuchenbaecker KB, Holmes MV, Engmann JE, Shah T, et al HMG-coenzyme A reductase inhibition, type 2 diabetes, and bodyweight: evidence from genetic analysis and randomised trials. Lancet. 2015;385(9965):351–61. 10.1016/S0140-6736(14)61183-1 25262344 PMC4322187

[9] Bocchetta A. Psychotic mania in glucose-6-phosphate-dehydrogenase-deficient subjects. Ann Gen Hosp Psychiatry. 2003;2(1):6 10.1186/1475-2832-2-6 12844366 PMC165592

[10] Heymann AD, Cohen Y, Chodick G. Glucose-6-phosphate dehydrogenase deficiency and type 2 diabetes. Diabetes care. 2012;35(8):e58 10.2337/dc11-2527 22826451 PMC3402279

[11] Niazi GA. Glucose-6-phosphate dehydrogenase deficiency and diabetes mellitus. International journal of hematology. 1991;54(4):295–8. . 1777604

[12] Saeed TK, Hamamy HA, Alwan AA. Association of glucose-6-phosphate dehydrogenase deficiency with diabetes mellitus. Diabet Med. 1985;2(2):110–2. . 2952393 10.1111/j.1464-5491.1985.tb00611.x

[13] Saha N. Association of glucose-6-phosphate dehydrogenase deficiency with diabetes mellitus in ethnic groups of Singapore. Journal of medical genetics. 1979;16(6):431–4. 537014 10.1136/jmg.16.6.431 PMC1012588

[14] Santana MS, Monteiro WM, Costa MR, Sampaio VS, Brito MA, Lacerda MV, et al High frequency of diabetes and impaired fasting glucose in patients with glucose-6-phosphate dehydrogenase deficiency in the Western brazilian Amazon. Am J Trop Med Hyg. 2014;91(1):74–6. 10.4269/ajtmh.13-0032 24865682 PMC4080572

[15] Pinna A, Contini EL, Carru C, Solinas G. Glucose-6-phosphate dehydrogenase deficiency and diabetes mellitus with severe retinal complications in a Sardinian population, Italy. International journal of medical sciences. 2013;10(13):1907–13. 10.7150/ijms.6776 24324368 PMC3856382

[16] Cocco P, Fadda D, Schwartz AG. Subjects expressing the glucose-6-phosphate dehydrogenase deficient phenotype experience a lower cardiovascular mortality. QJM. 2008;101(2):161–3. 10.1093/qjmed/hcm129 . 18160416

[17] Cocco P, Todde P, Fornera S, Manca MB, Manca P, Sias AR. Mortality in a cohort of men expressing the glucose-6-phosphate dehydrogenase deficiency. Blood. 1998;91(2):706–9. . 9427729

[18] Wiesenfeld SL, Petrakis NL, Sams BJ, Collen MF, Cutler JL. Elevated blood pressure, pulse rate and serum creatinine in Negro males deficient in glucose-6-phosphate dehydrogenase. N Engl J Med. 1970;282(18):1001–2. 10.1056/NEJM197004302821804 . 5436541

[19] Nwankwo MU, Bunker CH, Ukoli FA, Omene JA, Freeman DT, Vergis EN, et al Blood pressure and other cardiovascular disease risk factors in black adults with sickle cell trait or glucose-6-phosphate dehydrogenase deficiency. Genetic epidemiology. 1990;7(3):211–8. 10.1002/gepi.1370070305 . 2369999

[20] Heller P, Best WR, Nelson RB, Becktel J. Clinical implications of sickle-cell trait and glucose-6-phosphate dehydrogenase deficiency in hospitalized black male patients. N Engl J Med. 1979;300(18):1001–5. 10.1056/NEJM197905033001801 . 431593

[21] Bocchetta A, Piccardi MP, Del Zompo M. Is bipolar disorder linked to Xq28? Nature genetics. 1994;6(3):224 10.1038/ng0394-224 . 8012380

[22] Dern RJ, Glynn MF, Brewer GJ. Studies on the correlation of the genetically determined trait, glucose-6-phosphate dehydrogenase deficiency, with behavioral manifestations in schizophrenia. J Lab Clin Med. 1963;62:319–29. 14060461

[23] Mendlewicz J, Linkowski P, Wilmotte J. Linkage between glucose-6-phosphate dehydrogenase deficiency and manic-depressive psychosis. Br J Psychiatry. 1980;137:337–42. . 7448473 10.1192/bjp.137.4.337

[24] Del Zompo M, Bocchetta A, Goldin LR, Corsini GU. Linkage between X-chromosome markers and manic-depressive illness. Two Sardinian pedigrees. Acta psychiatrica Scandinavica. 1984;70(3):282–7. . 6333780 10.1111/j.1600-0447.1984.tb01210.x

[25] Baron M, Risch N, Hamburger R, Mandel B, Kushner S, Newman M, et al Genetic linkage between X-chromosome markers and bipolar affective illness. Nature. 1987;326(6110):289–92. 10.1038/326289a0 . 3493438

[26] Fieve RR, Brauninger G, Fleiss J, Cohen G. Glucose-6-phosphate dehydrogenase deficiency and schizophrenic behavior. J Psychiatr Res. 1965;3(4):255–62. . 5866614 10.1016/0022-3956(65)90006-3

[27] Baron M, Straub RE, Lehner T, Endicott J, Ott J, Gilliam TC, et al Bipolar disorder and linkage to Xq28. Nature genetics. 1994;7(4):461–2. 10.1038/ng0894-461a . 7951314

[28] Berrettini WH, Goldin LR, Gelernter J, Gejman PV, Gershon ES, Detera-Wadleigh S. X-chromosome markers and manic-depressive illness. Rejection of linkage to Xq28 in nine bipolar pedigrees. Arch Gen Psychiatry. 1990;47(4):366–73. . 2322087 10.1001/archpsyc.1990.01810160066010

[29] Baron M, Freimer NF, Risch N, Lerer B, Alexander JR, Straub RE, et al Diminished support for linkage between manic depressive illness and X-chromosome markers in three Israeli pedigrees. Nature genetics. 1993;3(1):49–55. 10.1038/ng0193-49 . 8490654

[30] Delisi LE, Crow TJ, Davies KE, Terwilliger JD, Ott J, Ram R, et al No genetic linkage detected for schizophrenia to Xq27-q28. Br J Psychiatry. 1991;158:630–4. . 1677599 10.1192/bjp.158.5.630

[31] Lam ST, Cheng ML. Neonatal screening in Hong Kong and Macau. Southeast Asian J Trop Med Public Health. 2003;34(Suppl 3):73–5. 15906700

[32] Schooling CM, Hui LL, Ho LM, Lam TH, Leung GM. Cohort profile: 'Children of 1997': a Hong Kong Chinese birth cohort. Int J Epidemiol. 2012;41(3):611–20. 10.1093/ije/dyq243 . 21224275

[33] World Health Organization, Department of Health of Hong Kong SAR. Health service delivery profile—Hong Kong (China) 2012. 2012.

[34] Wong CK. The Rutter Parent Scale A2 and Teacher Scale B2 in Chinese. II. Clinical validity among Chinese children. Acta psychiatrica Scandinavica. 1988;78(1):11–7. . 3176991 10.1111/j.1600-0447.1988.tb06295.x

[35] Battle J. Culture-free self-esteem inventories Austin, Texas: Pro-Ed; 1992.

[36] Lee W. Body dissatisfaction, depressive symptoms, and pubertal timing in Hong Kong Chinese Hong Kong: The University of Hong Kong; 2008.

[37] Fok TF, So HK, Wong E, Ng PC, Chang A, Lau J, et al Updated gestational age specific birth weight, crown-heel length, and head circumference of Chinese newborns. Arch Dis Child Fetal Neonatal Ed. 2003;88(3):F229–36. 10.1136/fn.88.3.F229 12719398 PMC1721552

[38] World Health Organization (WHO). WHO child growth standards Geneva, Switzerland: WHO; 2005.

[39] World Health Organization (WHO). WHO growth reference 5–19 years Geneva, Switzerland: WHO; 2007.

[40] National High Blood Pressure Education Program Working Group on High Blood Pressure in Children and Adolescents. The fourth report on the diagnosis, evaluation, and treatment of high blood pressure in children and adolescents. Pediatrics. 2004;114(2 Suppl 4th Report):555–76. . 15286277

[41] Peto R. Experimental survival curves for interval-censored data. Journal of the Royal Statistical Society Series C (Applied Statistics). 1973;22(1):86–91.

[42] Seaman SR, White IR, Copas AJ, Li L. Combining multiple imputation and inverse-probability weighting. Biometrics. 2012;68(1):129–37. 10.1111/j.1541-0420.2011.01666.x 22050039 PMC3412287

[43] Schafer JL. Multiple imputation: a primer. Stat Methods Med Res. 1999;8(3–15). 10.1177/096228029900800102 10347857

[44] Moons KG, Donders RA, Stijnen T, Harrell FE Jr. Using the outcome for imputation of missing predictor values was preferred. J Clin Epidemiol. 2006;59(10):1092–101. Epub 2006/09/19. S0895-4356(06)00060-6 [pii] 10.1016/j.jclinepi.2006.01.009 . 16980150

[45] Kwok MK, Freeman G, Lin SL, Lam TH, Schooling CM. Simulated growth trajectories and blood pressure in adolescence: Hong Kong's Chinese Birth Cohort. Journal of hypertension. 2013;31(9):1785–97. Epub 2013/06/12. 10.1097/HJH.0b013e3283622ea0 . 23751966

[46] Euling SY, Herman-Giddens ME, Lee PA, Selevan SG, Juul A, Sorensen TI, et al Examination of US puberty-timing data from 1940 to 1994 for secular trends: panel findings. Pediatrics. 2008;121 Suppl 3:S172–91. 10.1542/peds.2007-1813D . 18245511

[47] Utriainen P, Jaaskelainen J, Voutilainen R. Blood erythrocyte and hemoglobin concentrations in premature adrenarche. The Journal of clinical endocrinology and metabolism. 2013;98(1):E87–91. 10.1210/jc.2012-2783 . 23162102

[48] Lo KS, Wilson JG, Lange LA, Folsom AR, Galarneau G, Ganesh SK, et al Genetic association analysis highlights new loci that modulate hematological trait variation in Caucasians and African Americans. Human genetics. 2011;129(3):307–17. 10.1007/s00439-010-0925-1 21153663 PMC3442357

[49] Health Canada. Summary safety review—testosterone replacement products—cardiovascular risk 2014 [July 15, 2014]. Available: http://www.hc-sc.gc.ca/dhp-mps/medeff/reviews-examens/testosterone-eng.php.

[50] Ding EL, Song Y, Malik VS, Liu S. Sex differences of endogenous sex hormones and risk of type 2 diabetes: a systematic review and meta-analysis. JAMA. 2006;295(11):1288–99. 10.1001/jama.295.11.1288 . 16537739

[51] Cai X, Tian Y, Wu T, Cao CX, Li H, Wang KJ. Metabolic effects of testosterone replacement therapy on hypogonadal men with type 2 diabetes mellitus: a systematic review and meta-analysis of randomized controlled trials. Asian journal of andrology. 2014;16(1):146–52. Epub 2013/12/27. 10.4103/1008-682X.122346 24369149 PMC3901874

