# Supplementary material for: Glucose-6-Phosphate Dehydrogenase Deficiency and Physical and Mental Health until Adolescence
Source: PLoS One. 2016 Nov 8;11(11):e0166192. doi: 10.1371/journal.pone.0166192 (PMC5100951; doi:10.1371/journal.pone.0166192)
Supplement: S1 Appendix — (DOCX) [file pone.0166192.s001.docx]

S1 Appendix. R sample code for inverse probability weighting with multiple imputation (IPW/MI)

data <-read.csv("data.csv", sep=",", header=TRUE, fileEncoding="latin1")

# obs taking the value 1 if having information on G6PD status (exposure), 0 otherwise

include<-data[data$obs==1,]

exclude<-data[data$obs==0,]

### Step 1: Multiple imputation

# y – outcome, x1 – G6PD status (exposure), x2 – confounder, x3 - covariate

set.seed(100)

include.i <- aregImpute(~y+x1+x2+x3, data=include,n.impute=10)

include.nomiss <- list(include, include, include, include, include, include, include, include, include, include, include, include, include, include, include, include, include, include, include, include)

for(i in 1:20){

include.nomiss[[i]]$x2[is.na(include.nomiss[[i]]$x2)] <-

include.i$imputed$x2[,i]

include.nomiss[[i]]$x3[is.na(include.nomiss[[i]]$x3)] <-

include.i$imputed$x3[,i]

}

### Step 2: IPW weighting

# combine multiple imputed dataset + exclude dataset (without G6PD information)

merged.mi <- list(NA)

for(i in 1:20){

merged.mi[[i]]<-rbind(exclude,include.nomiss[[i]])

}

# exclude all missing from the combined dataset

merged <- list(NA)

for(i in 1:20){

merged[[i]]<-merged.mi[[i]][!is.na(merged.mi[[i]]$x2) & !is.na(merged.mi[[i]]$x3),]

}

# generate inverse probability weighting

missing.model<-list(NA)

for(i in 1:20){

missing.model[[i]]<-glm(obs~factor(x2) + factor(x3), data=merged[[i]], family=binomial)

merged[[i]]$pw<-(1/missing.model[[i]]$fitted.values)

}

### Step 3: association of G6PD status with outcome

analysis.model <- list(NA)

for(i in 1:20){

analysis.model[[i]] <- lm(y ~ x1+x2, weights=pw, data=merged[[i]])

}

# summarize results using Rubin’s rule

lm.combine.mi <- function(model, n.impute=20){

betas <- matrix(NA, ncol=20, nrow=length(model[[1]]$coef))

for (x in 1:20){

betas[,x]<-model[[x]]$coef

}

vars <- matrix(NA, ncol=20, nrow=length(model[[1]]$coef))

for (x in 1:20){

vars[,x] <- (summary(model[[x]])$coef[,2])^2

}

coef.names <- names(model[[1]]$coef)

mean.coefs <- rowMeans(betas)

Ubar <- rowMeans(vars)

B <- rowSums((betas - mean.coefs)*(betas-mean.coefs) /

(n.impute - 1))

T <- (1 + 1/n.impute) * B + Ubar

degf <- (n.impute - 1)*(1 + Ubar / ((1 + 1/n.impute)*B))*

(1 + Ubar / ((1 + 1/n.impute)*B))

data.frame(beta = mean.coefs,

lowerCI = mean.coefs - qt(0.975, df=degf)*sqrt(T),

upperCI = mean.coefs + qt(0.975, df=degf)*sqrt(T),

p.value = 2*(1 - pt(abs(mean.coefs)/sqrt(T), df=degf)),

row.names=coef.names)

}

# print results

combine.mi(analysis.model, n.impute=20)
