# Supplementary material for: Glucose-6-Phosphate Dehydrogenase Deficiency and Physical and Mental Health until Adolescence
Source: PLoS One. 2016 Nov 8;11(11):e0166192. doi: 10.1371/journal.pone.0166192 (PMC5100951; doi:10.1371/journal.pone.0166192)
Supplement: S1 Table — (DOCX) [file pone.0166192.s002.docx]

S1 Table. Baseline characteristics of 5,520 adolescents who were included in the analyses and 2,780 who were excluded from the analyses because of missing G6PD status in Hong Kong’s “Children of 1997” birth cohort, Hong Kong, China, 1997-2010

|  | Included  (*n*=5,520) | | Excluded  (*n*=2,780) | | Cohen effect size^b^ |
| --- | --- | --- | --- | --- | --- |
| Characteristics | No. | % | No. | % |  |
| Child’s sex |  |  |  |  | 0.05 |
| Female | 2,570 | 46.6 | 1,344 | 49.0 |  |
| Male | 2,949 | 53.4 | 1,401 | 51.0 |  |
| Mode of delivery |  |  |  |  | 0.17 |
| Natural labour | 2,844 | 53.7 | 1,651 | 62.1 |  |
| Assisted natural labour | 929 | 17.54 | 390 | 14.7 |  |
| Caesarean birth | 1,524 | 28.8 | 619 | 23.3 |  |
| Secondhand smoke exposure |  |  |  |  | 0.11 |
| None | 1,483 | 28.4 | 692 | 26.4 |  |
| Non-parental household smoking | 2,016 | 38.6 | 927 | 35.4 |  |
| Paternal smoking | 1,468 | 28.1 | 845 | 32.29 |  |
| Maternal smoking | 250 | 4.8 | 153 | 5.9 |  |
| Type of hospital at birth |  |  |  |  | 0.36 |
| Public | 3,649 | 67 | 2,040 | 84 |  |
| Private or overseas | 1,832 | 33.42 | 397 | 16.29 |  |
| Mother’s birthplace |  |  |  |  | 0.14 |
| Mainland China or elsewhere | 1,926 | 36.6 | 1,108 | 43.4 |  |
| Hong Kong | 3,341 | 63.4 | 1,445 | 56.6 |  |
| Highest parental education at recruitment |  |  |  |  | 0.15 |
| Grade 9 or below | 1,533 | 28.5 | 924 | 34.3 |  |
| Grade 10-11 | 2,293 | 42.7 | 1,148 | 42.6 |  |
| Grade 12 or above | 1,550 | 28.8 | 626 | 23.2 |  |
| Household income per head at recruitment ^a^ |  |  |  |  | 0.22 |
| 1^st^ quintile | 927 | 18.5 | 524 | 23.5 |  |
| 2^nd^ quintile | 955 | 19.1 | 529 | 23.8 |  |
| 3^rd^ quintile | 990 | 19.8 | 440 | 19.8 |  |
| 4^th^ quintile | 1,046 | 20.9 | 374 | 16.8 |  |
| 5^th^ quintile | 1,081 | 21.6 | 359 | 16.1 |  |
| Type of housing at recruitment |  |  |  |  | 0.16 |
| Public estate | 2,312 | 43.8 | 1,264 | 47.5 |  |
| Subsidized home ownership flat | 851 | 16.1 | 275 | 10.3 |  |
| Private flat | 2,122 | 40.2 | 1,124 | 42.2 |  |

^a^ Mean (standard deviation) for household income per head at recruitment in quintiles (in Hong Kong dollar; pegged at a rate of 7.8 dollar = 1 U.S. dollar) were 1^st^ quintile: $1,745 (419), 2^nd^ quintile: $2,853 (325), 3^rd^ quintile: $4,367 (557), 4^th^ quintile: $6,826 (883) and 5^th^ quintile: $14,943 (15,635)

^b^ Cohen effect sizes are usually categorized into 3 levels: 0.1 for small, 0.3 for medium, and 0.5 for large. For categorical variables, Cohen effect size are calculated as √(Σ(p1i-p2i)^2^/p1i) where the sum is over the categories and p0i is the proportion in the ith category in the included subjects and p2i is the proportion in the ith category in the excluded subjects.
