# Supplementary material for: Glucose-6-Phosphate Dehydrogenase Deficiency and Physical and Mental Health until Adolescence
Source: PLoS One. 2016 Nov 8;11(11):e0166192. doi: 10.1371/journal.pone.0166192 (PMC5100951; doi:10.1371/journal.pone.0166192)
Supplement: S2 Table — (DOCX) [file pone.0166192.s003.docx]

S2 Table. Adjusted^a^ association of G6PD status with birth weight-for-gestational age z-score for growth during fetal phase, height and body mass index (BMI) gain z-scores during infancy, childhood and pubertal phases, age at onset of breast or genitalia or pubic hair development (Tanner stage II), testicular volume, age at menarche and blood pressure at ~11 and ~13 years, height and BMI z-scores at ~15 years among boys in the Hong Kong’s “Children of 1997” birth cohort, Hong Kong, China, 1997-2010

| Age | Outcomes | G6PD status | n | Mean difference^c^ | 95% CI | Follow-up n (%) |
| --- | --- | --- | --- | --- | --- | --- |
| Fetal | Birth weight-for- | Deficient | 140 | -0.02 | -0.18, 0.14 | 2,948 |
|  | gestational age z-score | Non-deficient | 2,808 | Reference |  | (100.0) |
| Infancy | Length gain z-score^b^ | Deficient | 115 | 0.03 | -0.12, 0.18 | 2,445 |
|  |  | Non-deficient | 2,330 | Reference |  | (82.9) |
|  | BMI gain z-score^b^ | Deficient | 115 | -0.01 | -0.20, 0.19 | 2,445 |
|  |  | Non-deficient | 2,330 | Reference |  | (82.9) |
| Childhood | Height gain z-score^b^ | Deficient | 126 | -0.02 | -0.15, 0.11 | 2,698 |
|  |  | Non-deficient | 2,572 | Reference |  | (91.5) |
|  | BMI gain z-score^b^ | Deficient | 126 | -0.39 | -0.58, -0.20 | 2,698 |
|  |  | Non-deficient | 2,572 | Reference |  | (91.5) |
| Puberty | Height gain z-score^b^ | Deficient | 130 | -0.06 | -0.13, -0.004 | 2,762 |
|  |  | Non-deficient | 2,632 | Reference |  | (93.7) |
|  | BMI gain z-score^b^ | Deficient | 130 | -0.05 | -0.18, 0.08 | 2,762 |
|  |  | Non-deficient | 2,632 | Reference |  | (93.7) |
|  |  |  | n | Time ratio | 95% CI |  |
|  | Age at onset of breast or | Deficient | 123 | 1.008 | 0.987, 1.030 | 2,739 |
|  | genitalia development | Non-deficient | 2,616 | 1.000 |  | (92.9) |
|  | Age at onset of pubic | Deficient | 125 | 1.036 | 1.014, 1.058 | 2,761 |
|  | hair development | Non-deficient | 2,636 | 1.000 |  | (93.6) |
|  | Age at onset of testes | Deficient | 124 | 1.008 | 0.988, 1.030 | 2,743 |
|  | development | Non-deficient | 2,619 | 1.000 |  | (93.0) |
|  |  |  | n | Mean difference^c^ | 95% CI |  |
|  | Testicular size | Deficient | 125 | -0.22 | -0.74, 0.30 | 2,763 |
|  |  | Non-deficient | 2,638 | Reference |  | (93.7) |
|  |  |  |  |  |  |  |
| 11 years | Systolic blood pressure | Deficient | 111 | -0.01 | -0.19, 0.17 | 2,366 |
|  | z-score | Non-deficient | 2,255 | Reference |  | (80.2) |
|  | Diastolic blood pressure | Deficient | 111 | 0.03 | -0.21, 0.26 | 2,366 |
|  | z-score | Non-deficient | 2,255 | Reference |  | (80.2) |
|  |  |  |  |  |  |  |
| 13 years | Systolic blood pressure | Deficient | 89 | 0.06 | -0.04, 0.16 | 2,015 |
|  | z-score | Non-deficient | 1,926 | Reference |  | (68.3) |
|  | Diastolic blood pressure | Deficient | 89 | -0.04 | -0.15, 0.08 | 2,015 |
|  | z-score | Non-deficient | 1,926 | Reference |  | (68.3) |
| 15 years | Height z-score | Deficient | 69 | -0.03 | -0.24, 0.17 | 1,602 |
|  |  | Non-deficient | 1,533 | Reference |  | (54.3) |
|  | BMI z-score | Deficient | 69 | -0.14 | -0.46, 0.18 | 1,602 |
|  |  | Non-deficient | 1,533 | Reference |  | (54.3) |

^a^ Adjusted for highest parental education

^b^ Additionally adjusted for initial size (birth weight z-score for infancy, height or BMI z-score at 9 months for childhood phase, height or BMI z-score at 7 years for pubertal phase)

^c^ Mean difference in z-score: 1 unit change in birth weight-for-gestational age z-score is approximated to 370 grams; 1 unit change in height z-score is approximated to 2.3 cm at 9 months, 5.6 cm at 7 years and 7.4 cm at 13 years ; 1 unit change in body mass index z-score is approximated to 1.5 kg/m^2^ at 9 months, 1.9 kg/m^2^ at 7 years and 2.7 kg/m^2^ at 13 years; 1 unit change in systolic blood pressure z-score is approximated to 10.6 mmHg and 1 unit change in diastolic blood pressure z-score is approximated to 11.3 mmHg.
