# Supplementary material for: Glucose-6-Phosphate Dehydrogenase Deficiency and Physical and Mental Health until Adolescence
Source: PLoS One. 2016 Nov 8;11(11):e0166192. doi: 10.1371/journal.pone.0166192 (PMC5100951; doi:10.1371/journal.pone.0166192)
Supplement: S3 Table — (DOCX) [file pone.0166192.s004.docx]

S3 Table. Adjusted^a^ association of G6PD status with time to first hospitalization for respiratory infections, gastrointestinal infections, other (non-respiratory or non-gastrointestinal) infections and all infections up until 12 years by age groups among boys in the Hong Kong’s “Children of 1997” birth cohort, Hong Kong, China, 1997-2010

| Age | Hospitalization | G6PD status | Case no.  n (%) | Hazard ratios | 95% CI |
| --- | --- | --- | --- | --- | --- |
| 9 days to 12 years |  |  |  |  |  |
|  | Respiratory infections | Deficient | 37 (24.2%) | 0.90 | 0.68, 1.19 |
|  |  | Non-deficient | 1,368 (25.5%) | 1.00 |  |
|  | Gastrointestinal infections | Deficient | 17 (11.1%) | 0.86 | 0.56, 1.31 |
|  |  | Non-deficient | 613 (11.4%) | 1.00 |  |
|  | Other infections | Deficient | 17 (11.1%) | 0.84 | 0.55, 1.28 |
|  |  | Non-deficient | 600 (11.2%) | 1.00 |  |
|  | All infections | Deficient | 56 (36.6%) | 0.92 | 0.73, 1.16 |
|  |  | Non-deficient | 1,992 (37.1%) | 1.00 |  |
|  |  |  |  |  |  |
| 9 days to 6 years |  |  |  |  |  |
|  | Respiratory infections | Deficient | 32 (20.9%) | 0.84 | 0.62, 1.14 |
|  |  | Non-deficient | 1,262 (23.5%) | 1.00 |  |
|  | Gastrointestinal infections | Deficient | 16 (10.5%) | 0.85 | 0.55, 1.32 |
|  |  | Non-deficient | 574 (10.7%) | 1.00 |  |
|  | Other infections | Deficient | 16 (10.5%) | 0.86 | 0.55, 1.33 |
|  |  | Non-deficient | 542 (10.1%) | 1.00 |  |
|  | All infections | Deficient | 52 (34.0%) | 0.91 | 0.71, 1.16 |
|  |  | Non-deficient | 1,855 (34.6%) | 1.00 |  |
| 6 to <12 years |  |  |  |  |  |
|  | Respiratory infections | Deficient | 8 (5.2%) | 1.30 | 0.67, 2.51 |
|  |  | Non-deficient | 201 (3.8%) | 1.00 |  |
|  | Gastrointestinal infections | Deficient | 3 (2.0%) | 1.82 | 0.42, 7.90 |
|  |  | Non-deficient | 52 (1.0%) | 1.00 |  |
|  | Other infections | Deficient | 2 (1.3%) | 1.44 | 0.39, 5.39 |
|  |  | Non-deficient | 69 (1.3%) | 1.00 |  |
|  | All infections | Deficient | 13 (8.5%) | 1.18 | 0.72, 1.95 |
|  |  | Non-deficient | 303 (5.7%) | 1.00 |  |

^a^ Adjusted for highest parental education and proxies of preferred service sector (type of hospital at birth and household income per head)
