# Supplementary material for: Glucose-6-Phosphate Dehydrogenase Deficiency and Physical and Mental Health until Adolescence
Source: PLoS One. 2016 Nov 8;11(11):e0166192. doi: 10.1371/journal.pone.0166192 (PMC5100951; doi:10.1371/journal.pone.0166192)
Supplement: S4 Table — (DOCX) [file pone.0166192.s005.docx]

S4 Table. Adjusted^a^ association of G6PD status with mental health indicated by mean difference in Rutter score at ~11 years, self-esteem score at ~11 years and depressive symptoms score at ~13 years among boys in the Hong Kong’s “Children of 1997” birth cohort, Hong Kong, China, 1997-2010

| Age | Outcomes | G6PD status | n | Mean difference | 95% CI | Follow-up n (%) |
| --- | --- | --- | --- | --- | --- | --- |
| 11 years | **Rutter scores** |  |  |  |  | 2,018 |
|  | Total | Deficient | 96 | -0.04 | -0.17, 0.08 | (68.4) |
|  |  | Non-deficient | 1,922 | Reference |  |  |
|  | Conduct | Deficient | 96 | -0.002 | -0.16, 0.15 |  |
|  |  | Non-deficient | 1,922 | Reference |  |  |
|  | Emotional | Deficient | 96 | -0.02 | -0.19, 0.16 |  |
|  |  | Non-deficient | 1,922 | Reference |  |  |
|  | Hyperactivity | Deficient | 96 | -0.14 | -0.31, 0.04 |  |
|  |  | Non-deficient | 1,922 | Reference |  |  |
|  |  |  |  |  |  |  |
| 11 years | **Self-esteem scores** |  |  |  |  | 2,486 |
|  | Total | Deficient | 115 | 0.004 | -0.03, 0.04 | (84.3) |
|  |  | Non-deficient | 2,371 | Reference |  |  |
|  | General | Deficient | 115 | 0.002 | -0.04, 0.05 |  |
|  |  | Non-deficient | 2,371 | Reference |  |  |
|  | Social | Deficient | 115 | -0.01 | -0.08, 0.05 |  |
|  |  | Non-deficient | 2,371 | Reference |  |  |
|  | Academic | Deficient | 115 | 0.02 | -0.04, 0.08 |  |
|  |  | Non-deficient | 2,371 | Reference |  |  |
|  | Parent-related | Deficient | 115 | 0.01 | -0.05, 0.07 |  |
|  |  | Non-deficient | 2,371 | Reference |  |  |
| 13 years | **Depressive symptoms** |  |  |  |  | 2,085 |
|  | PHQ-9 score | Deficient | 100 | 0.06 | -0.14, 0.26 | (70.7) |
|  |  | Non-deficient | 1,985 | Reference |  |  |

^a^ Adjusted for highest parental education, age at measurement and survey mode (for PHQ-9)
